# Prevalence of Hypertension in Member Countries of South Asian Association for Regional Cooperation (SAARC): Systematic Review and Meta-Analysis

**DOI:** 10.1097/MD.0000000000000074

**Published:** 2014-09-12

**Authors:** Dinesh Neupane, Craig S. McLachlan, Rajan Sharma, Bishal Gyawali, Vishnu Khanal, Shiva Raj Mishra, Bo Christensen, Per Kallestrup

**Affiliations:** Department of Public Health (DN, PK), Center for Global Health, Aarhus University, Aarhus, Denmark; Rural Clinical School (CSM), University of New South Wales, Sydney, Australia; Unit of Health Promotion (RS, BG), University of Southern Denmark, Esbjerg, Denmark; School of Public Health (VK), Curtin University, Perth, Australia; Nepal Development Society (SRM), Kathmandu, Nepal; and Department of Public Health (BC), Institute of General Medical Practice, Aarhus University, Aarhus, Denmark.

## Abstract

Hypertension is a leading attributable risk factor for mortality in South Asia. However, a systematic review on prevalence and risk factors for hypertension in the region of the South Asian Association for Regional Cooperation (SAARC) has not carried out before.

The study was conducted according to the Meta-Analysis of Observational Studies in Epidemiology Guideline. A literature search was performed with a combination of medical subject headings terms, “hypertension” and “Epidemiology/EP”. The search was supplemented by cross-references. Thirty-three publications that met the inclusion criteria were included in the synthesis and meta-analyses. Hypertension is defined when an individual had a systolic blood pressure (SBP) ≥140 mm Hg and/or diastolic blood pressure (DBP) ≥90 mm Hg, was taking antihypertensive drugs, or had previously been diagnosed as hypertensive by health care professionals. Prehypertension is defined as SBP 120–139 mm Hg and DBP 80–89 mm Hg.

The overall prevalence of hypertension and prehypertension from the studies was found to be 27% and 29.6%, respectively. Hypertension varied between the studies, which ranged from 13.6% to 47.9% and was found to be higher in the studies conducted in urban areas than in rural areas. The prevalence of hypertension from the latest studies was: Bangladesh: 17.9%; Bhutan: 23.9%; India: 31.4%; Maldives: 31.5%; Nepal: 33.8%; Pakistan: 25%; and Sri Lanka: 20.9%. Eight out of 19 studies with information about prevalence of hypertension in both sexes showed that the prevalence was higher among women than men. Meta-analyses showed that sex (men: odds ratio [OR] 1.19; 95% confidence interval [CI]: 1.02, 1.37), obesity (OR 2.33; 95% CI: 1.87, 2.78), and central obesity (OR 2.16; 95% CI: 1.37, 2.95) were associated with hypertension.

Our study found a variable prevalence of hypertension across SAARC countries, with a number of countries with blood pressure above the global average. We also noted that studies are not consistent in their data collection about hypertension and related modifiable risk factors.

## INTRODUCTION

Hypertension has reached epidemic proportions worldwide and significantly contributes to the burden of heart disease, stroke, kidney failure, disability, and premature death.^1^ It is estimated that about 17 million deaths occur worldwide because of cardiovascular diseases (CVDs) every year, of which complications of hypertension alone account for 9.4 million deaths.^2^ Although the rate of hypertension is rising in the developed countries such in the USA,^3^ the rate of increase is faster in many low and middle-income countries.^1^ The risk factors for hypertension include aging populations and adverse changes in risk factors such as tobacco use, low physical activity, and unhealthy diet, especially high salt consumption.^4^ The South Asian Association for Regional Cooperation (SAARC) region is a home to almost one-quarter of the world’s population and is comprised of diverse ethnic, linguistic, and religious groups. India, Pakistan, Bangladesh, Nepal, Afghanistan, Sri Lanka, Bhutan, and Maldives are the countries of this region, which are the members of SAARC. Despite considerable diversity among the inhabitants of these countries, there are similarities in the sociocultural aspects of the people from this region. According to the World Health Organisation (WHO) estimates, hypertension has become an important health concern in the Asian region affecting more than 35% of the adult population.^5^ Recent WHO estimates show that the prevalence of hypertension in the SAARC region appears to be higher,^6^ although this has not yet been documented collectively such as through a systematic review and meta-analysis. Furthermore, in most of the SAARC member states, there are no national guidelines for the prevention and control of hypertension.^4^ The purpose of this review is to estimate the prevalence of hypertension reported among adults across SAARC countries and to investigate associated risk factors reported by such studies.

## METHODS

**FIGURE 1:**
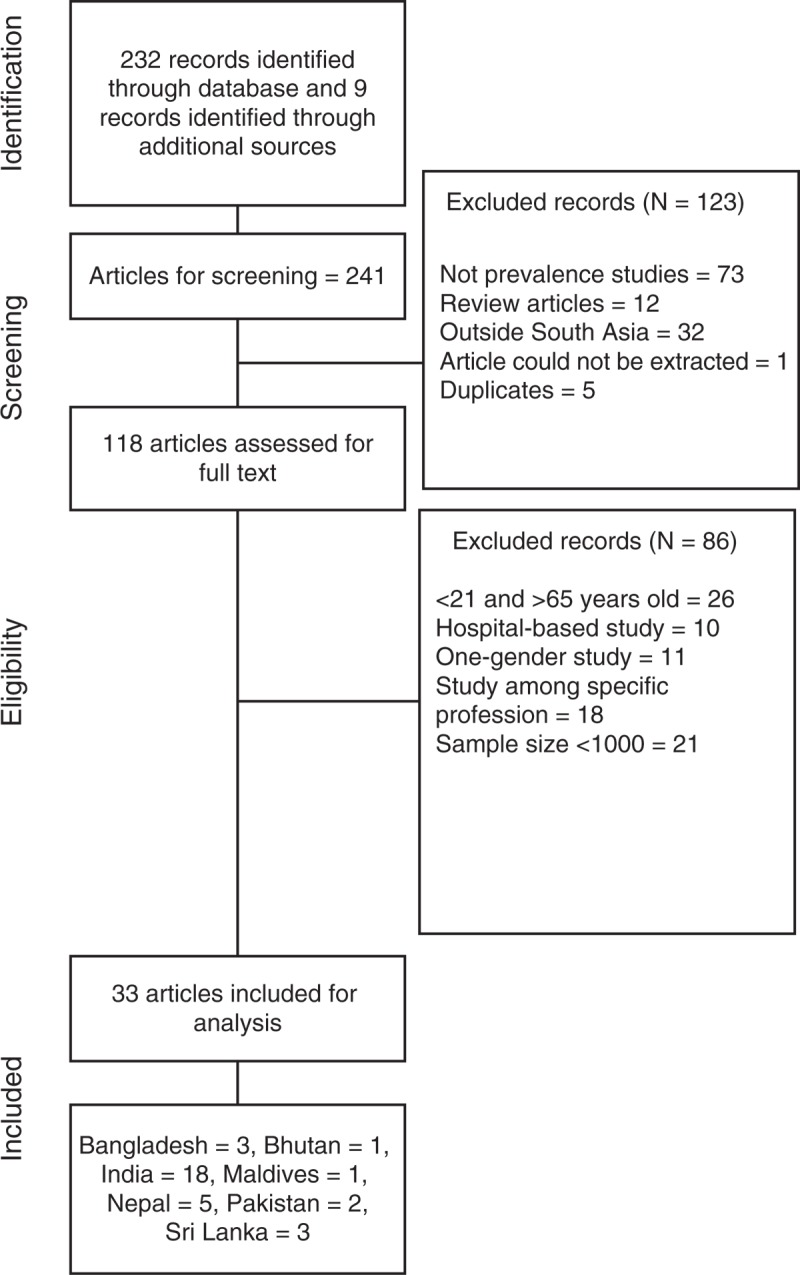
Flow diagram of study.

The study was conducted according to the Meta-Analysis of Observational Studies in Epidemiology Guideline.^7^ Ethical approval for systematic reviews remains a gray area.^8^ We did not seek ethical approval, although we did review each article as to whether ethical approval was obtained; 54% of the articles described institution ethics approval and 54% reported that individual consent was obtained. Fifteen percent of the articles mentioned that written informed consent was obtained. From the initially identified 240 articles, a total of 33, which met our inclusion criteria, were included for further analysis. The detailed exclusion and inclusion criteria as well as extraction process of the articles are described further in the text and also diagrammatically illustrated in Figure 1.

Data were extracted through a 3-stage selection process. In the first stage, a search of the online Medical Literature Analysis and Retrieval System (MEDLINE) database was performed with a combination of medical subject headings (MeSH) terms: “hypertension” as the MeSH major topic and “Epidemiology/EP” as the MeSH subheading. We did not use “prehypertension” as a MeSH term because this search term was only introduced in 2011 and definitions of prehypertension cannot be ascertained from studies predating 2011.

The search limits were: language (“English”), dates (between “1st January 2000” and “30th September 2013”), and species (“Humans”). Furthermore, the result was narrowed down adding the name of each SAARC member country (Afghanistan, Bangladesh, Bhutan, India, Nepal, Maldives, Pakistan, and Sri Lanka) and South Asia as keywords. We also performed a manual search for other articles in WHO publications obtained from the WHO website and references of published articles. We also attempted to contact some authors for article extraction. Only 1 article was retrieved by directly consulting the corresponding author.

In the second stage, titles and abstracts were reviewed using our predefined screening criteria. If the required information was not available in the abstract, full-text articles were further reviewed to gather the information. Studies not satisfying the inclusion criteria were excluded at this stage. Studies conducted outside SAARC countries, nonoriginal articles such as review articles and letters to the editor and not from population-based cross-sectional studies, were excluded. In the third stage, an exclusion criteria was used for further filtering of the articles including only specific age (<21 and >65 years), only 1 gender reported on, hospital-based studies, studies among specific sectors of population such as bank employees or tea workers, and a sample size <1000. The reason behind excluding studies conducted before 2000 was to include hypertension definitions and measurements conducted according to the WHO revised definition in the year 1999.^9^ Similarly, as per EPI INFO (Version 7, Center for Disease Control, Atlanta, GA), a sample size of 400 selected through simple random sampling is adequate to detect prevalence of hypertension between 10% and 50% with 5% precision and 95% confidence. However, we did not include studies having sample size less than 1000 considering the potential nonresponse rate and design effect used by individual studies. Therefore, considering design effect assumed to be 2 and expected response rate of 80%, the minimum sample size for detecting prevalence would be 960. So, we have included only those studies having sample size of at least 1000 or more. In addition to this, we have excluded studies that “only” focused on populations >65 and <15 years. The rationale is that the studies conducted among adults aged >65 years have been found to report higher prevalence rates (particularly because of systolic stiffening). Studies conducted focusing on participants <15 years would not be representative of the average hypertension prevalence rates for adult populations. Thus, we have included studies that have included broad age ranges including adults of any age, including >65 age group and/or in addition to teenage age groups if these population age ranges were included in our systematic review criteria (see text below). As part of our search strategy, we selected only population-based cross-sectional surveys estimating the prevalence of hypertension, in both genders, a broad age group, not restricted in specific profession, conducted in member states of SAARC, having sample size more than 1000, published in English language, and in the period from January 1, 2000 to September 30, 2013.

Data from Afghanistan were not included in further analysis. Only the latest WHO publications from stepwise approach to surveillance (STEP) were included in our analyses. India and Pakistan did not have WHO STEP surveys available, whereas a scientific article was published from Maldives’s STEP survey. In summary, the last edition of the STEP survey from Nepal, Bangladesh, Sri Lanka, and Bhutan were included for the secondary data analysis.

The characteristics recorded for each study included first author’s name, year of publication, country of origin, sampling methods, characteristics of the participants (age, sex), sample size, methods of blood pressure (BP) measurement (type of device, number of BP readings taken, and time interval between the measurements), definition of hypertension, response rate, prevalence of hypertension, location of study (rural/urban), and mean systolic blood pressure (SBP) and diastolic blood pressure (DBP). Wherever available, odds ratio (OR) with respective confidence interval (CI) and adjusted variables for the associated risk factors (men, obesity, central obesity, smoking, low physical activity, diabetes, family history, high salt intake, low fruit and vegetable intake, alcohol intake, literacy, high fat intake, high triglyceride, and high cholesterol) were recorded.

Hypertension is defined as SBP ≥140 mm Hg and/or DBP ≥90 mm Hg, taking antihypertensive drugs, or previously diagnosed by health care workers, and prehypertension is defined as SBP 120–139 mm Hg and DBP 80–89 mm Hg unless stated specifically. We have not included studies that included participants who self-reported BP measurement. Only 2 studies have been conducted in Asia on self-reported BP (Taiwan and Thailand) and therefore did not meet our search criteria.^10^ The pooled prevalence was calculated by using standard error of prevalence that is given by √[p ×  (1 − p)/n], where p is the proportion of prevalence and n is the reported sample size.

The rural and urban categories were made based on the information provided by the authors. We also considered the possibility of different cutoff values used for the categorization of risk factors for hypertension. To adjust for more than 2 categories, we calculated pooled OR. Meta-analysis was performed for a minimum of 5 articles, which reported OR. After assessing the heterogeneity by calculating *I*^2^ (% residual variation because of heterogeneity) and τ^2^ (method of moments estimate of between-study variance) for each of the pooled estimates, random effect meta-analysis was carried out for detected significant heterogeneity (*P* < 0.001 from χ^2^ test). Data were analyzed using STATA 13 (StataCorp, College Station, TX).

## RESULTS

### Study Selection

We identified 33 published studies based on our methodology presented in the previous section. The summary of the study characteristics from SAARC countries is presented in Table 1. Our search did not yield any publications from Afghanistan. According to the WHO estimates, the crude prevalence of hypertension among persons aged 25 or above in 2008 in Afghanistan was 31.9%.^11^ The majority of retrieved articles were from India (N = 18) followed by Nepal (N = 5), Pakistan (N = 2), Bangladesh (N = 3), Sri Lanka (N = 3), Maldives (N = 1), and Bhutan (N = 1).

**TABLE 1:**
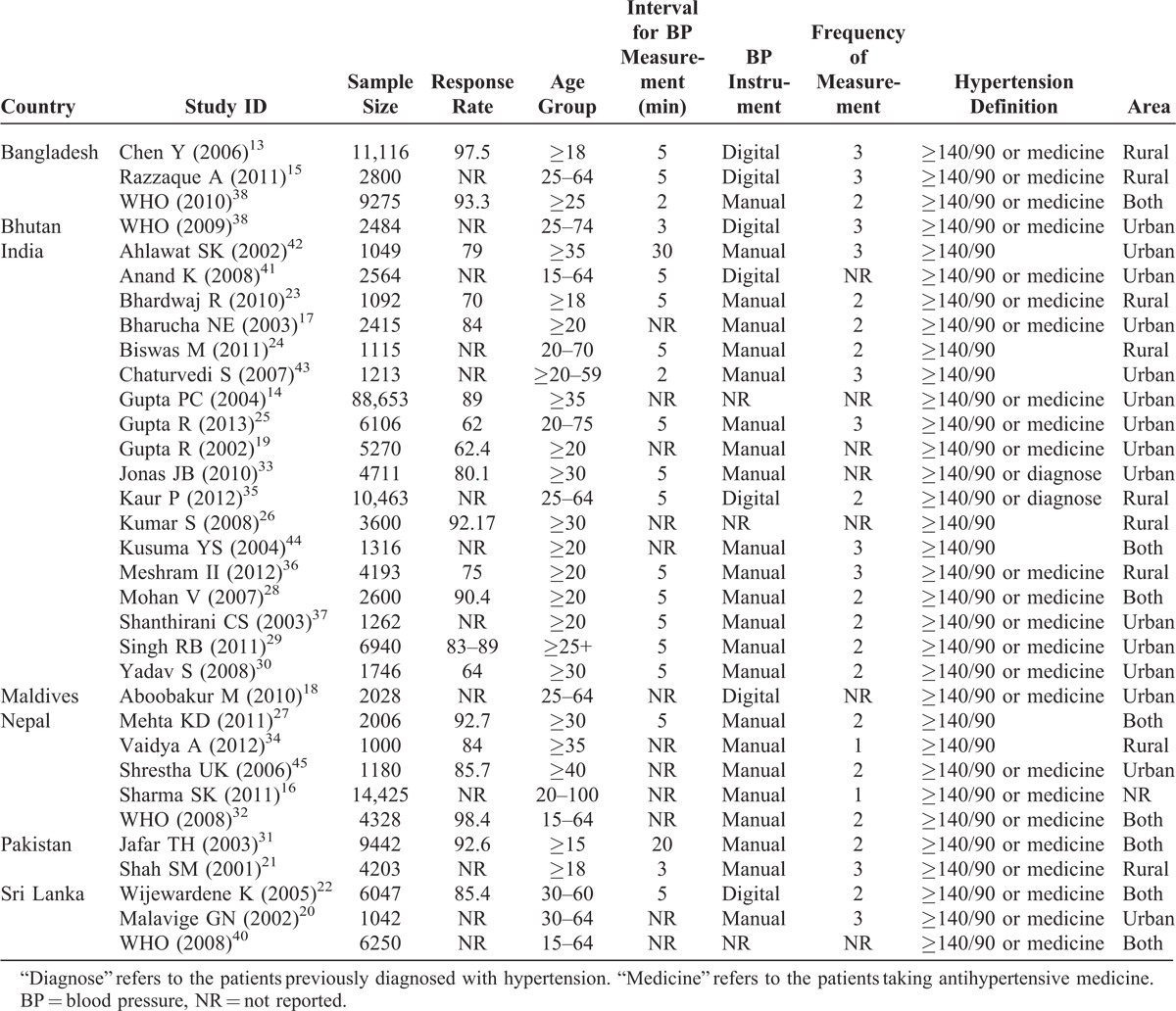
Study Characteristics

### Study Characteristics

The sample size ranged from 1012 to 88,653. The total sample size included for this review was 220,539. The response rate varied from 62% to 98%. The highest numbers of studies (N = 6) were published in 2011 followed by 2008 (N = 5). Most of the studies were conducted among participants over 20 years of age. The overall mean age of participants was 43.65 years (standard deviation [SD] = 5.92) ranging from 28.19 to 54.54 years. A total of 9 studies used multistage sampling followed by stratified (N = 4) sampling methods. Of all studies, 22 used manual sphygmomanometers, 8 used digital sphygmomanometers, and 3 studies did not provide information about the measuring devices. The range of frequency of BP measurements varied from 1 to 3 and intervals between each measurement ranged between 2 minutes and half an hour.

### Data Quality

The quality of the studies was evaluated by developing a modified strengthening the reporting of observational studies in epidemiology checklist.^12^ Articles were assigned high and low scores for the selection procedure of participants, frequency of measurement of hypertension, and response rates. Studies using random sampling, having a high response rate (>70%), taking more than 1 measurement, and those clearly explaining the limitations of the study were assigned a high score. Only 4 articles fulfilled the criteria for being high-quality articles. Majority of the articles did not report the limitations of their studies. Although quality was rated for each study, quality scores were not incorporated in the meta-analysis weights.

### Burden of Hypertension

**FIGURE 2:**
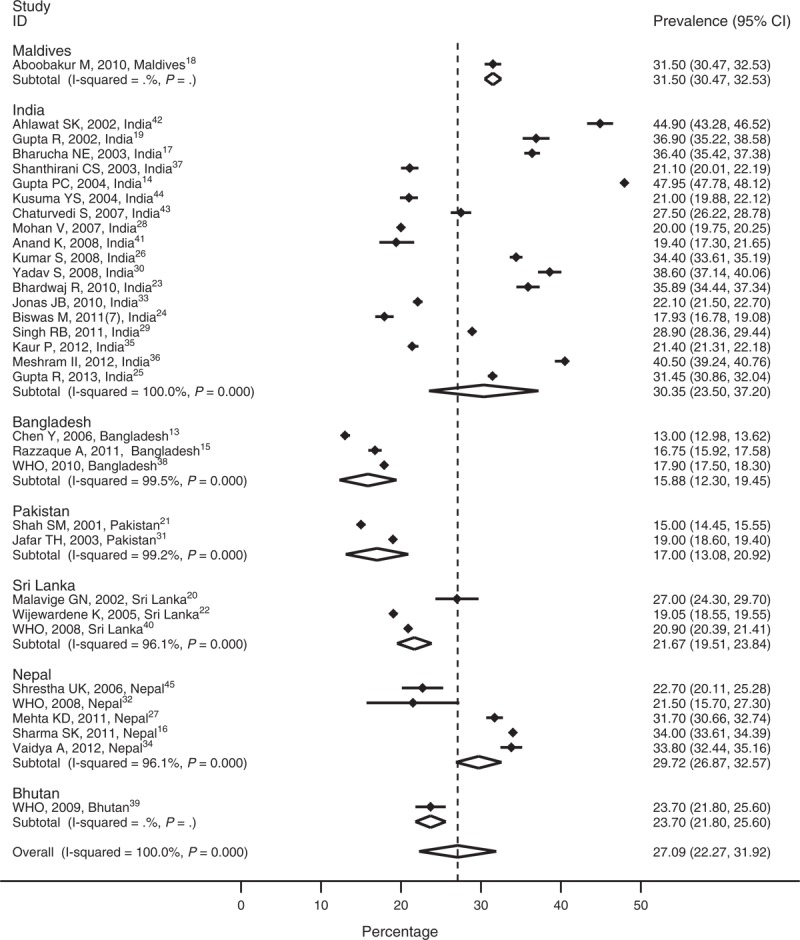
Meta-analysis of prevalence of hypertension.

The prevalence of hypertension found in the studies is presented in Figure 2. There was a considerable heterogeneity in the prevalence of hypertension, depending on where the research was conducted and which study design was employed. The prevalence of hypertension ranged from 13% to 47%.^13,^ The mean prevalence of hypertension was found to be 27%. It is important to note that 13 out of 33 studies reported prevalence higher than 30%. The mean prevalence reported in urban areas (N = 14) was 31.2% and in rural areas (N = 9) was 24%.

The average prevalence of hypertension in men and women was 27% (SD = 9.90) and 25% (SD = 9.58), respectively. The prevalence among urban areas for men (N = 9) was 31% (SD = 7.15) and for women (N = 10) was 31% (SD = 9.21). In rural areas (N = 4), the prevalence for men was 22% (SD = 15.49) and for women was 21% (SD = 10.17).

The lowest and the highest prevalence for men and women was reported from the study conducted in Bangladesh^15^ and India,^14^ respectively. The reported maximum and minimum differences in prevalence rates among men and women were from Nepal (10.7%)^16^ and India (−6.6%),^17^ respectively. Out of 19 studies that reported prevalence of hypertension for both sexes, 8 studies (3 from India, 1 from Bangladesh, 2 from Sri Lanka, 1 from Pakistan, and 1 from Maldives) reported higher prevalence for women when compared with men.^14,15,17–22^

Nine studies reported the prevalence of prehypertension.^13,23–30^ The overall prevalence of prehypertension from these studies was 29%. The prevalence of prehypertension was reported lowest from an Indian study (17%)^24^ and highest from a Nepali study (38%)^27^ using similar cutoff points. Prehypertension prevalence was found to be much higher in the urban areas (mean = 32%, range = 28%–35%) as compared with rural areas (mean = 23%, range = 17%–28%).

### Systolic and Diastolic Blood Pressure

The reported SBP and DBP are presented in Table 2. The highest mean SBP and DBP were reported from a study conducted in Nepal (138.72/93.09 mm Hg)^16^ whereas the lowest mean SBP and DBP were reported from the studies conducted in Pakistan (118.80 mm Hg)^31^ and Sri Lanka (74.40 mm Hg), respectively.^22^ Only 17 studies reported mean SBP and DBP for men and women separately.

**TABLE 2:**
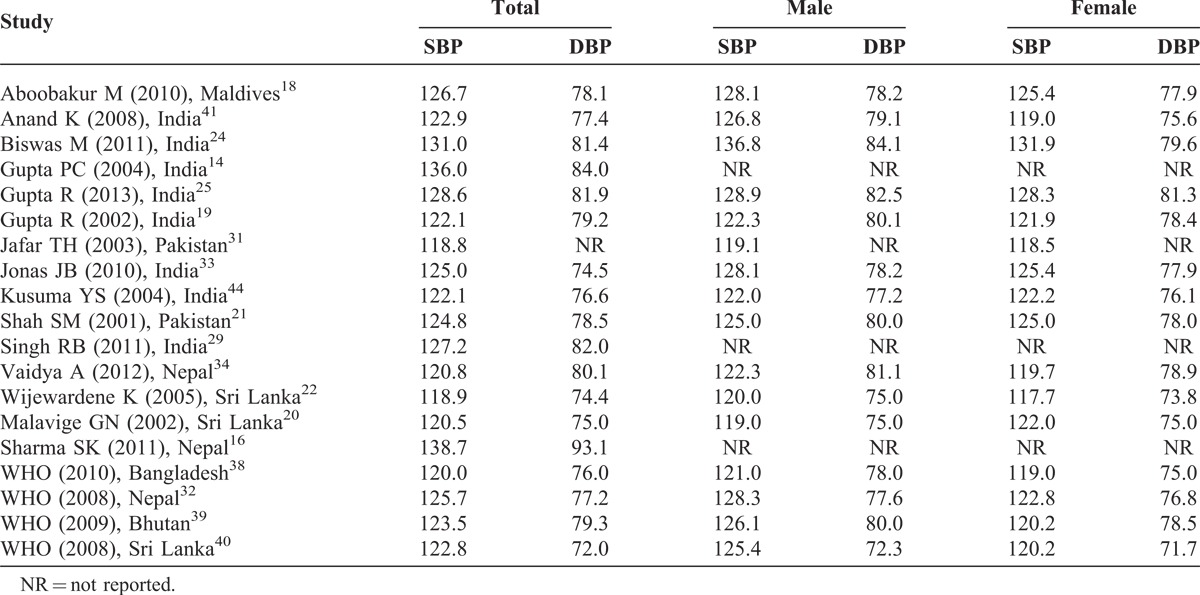
Systolic and Diastolic Blood Pressure

Although the overall correlation between published year and mean SBP and DBP (SBP: *r* = 0.279, N = 25; DBP: *r* = 0.118, N = 24) was weak, we found that only 1 study^14^ reported mean SBP ≥125 mm Hg before 2006, but there were 7 studies^16,18,24,25,29,32,33^ that reported ≥125 mm Hg after 2006. The trend was also similar for DBP. There was only 1 study^14^ reporting more than 80 mm Hg mean BP before 2006 compared with 5 studies^16,24,25,29,34^ reported after 2006.

### Risk Factors

Heterogeneity was observed in the classification of different risk factors. A study^25^ conducted in India categorized education as <10, 10–15, and >15 years whereas another study^35^ used 4 different categories of education: achieved 5 years of schooling, 8 years of schooling, 12 years of schooling, and college-level education. Similarly, the categorization of physical activity was also inconsistent. Out of 6 studies used for meta-analysis, 1 study^34^ categorized no regular physical exercise as sedentary life; based on occupation, 1 study^36^ categorized housewives, landlords, business, and pensions as sedentary life. Remaining 4 studies^25,29,30,37^ classified low physical activities based on noninvolvement in any work or leisure time-related physical activities. Regarding tobacco use, 2 studies^28,37^ used current smoker or nonsmoker category, 1 study^25^ used the WHO criteria, 1 study^35^ used passive, active, and current smoker categories, and 1 study^34^ did not mention the classification category. The heterogeneity in classification was also found for both overweight and central obesity. Regarding obesity, 3 studies^30,35,36^ used ≥27.5 kg/m^2^, 2 studies^25,28^ used ≥25 kg/m^2^, and 1 study^34^ used ≥23 kg/m^2^. Regarding central obesity, 3 studies^28,35,36^ used ≥90 cm for men and ≥80 cm for women, 1 study^25^ used >100 cm for men and >90 cm for women, 1 study^34^ used waist hip ratio 0.88 for men and 0.81 for women, and 1 study^34^ did not specify the cutoff. Adjusted variables are presented in Table 3. Two studies^30,37^ reported univariate analysis whereas most of the studies adjusted for age, body mass index, and gender.

**TABLE 3:**
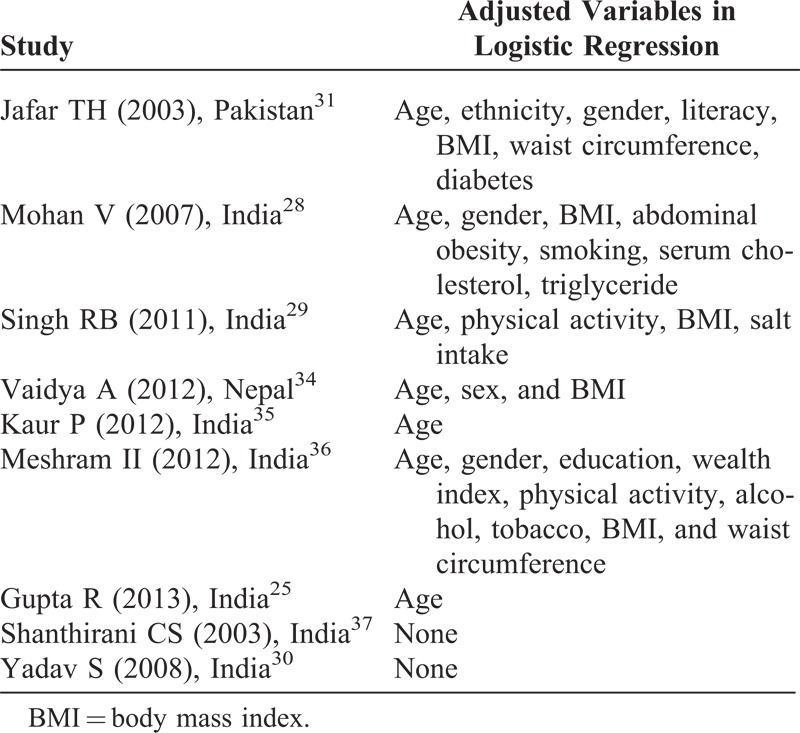
Adjusted Variables in Logistic Regression

**FIGURE 3:**
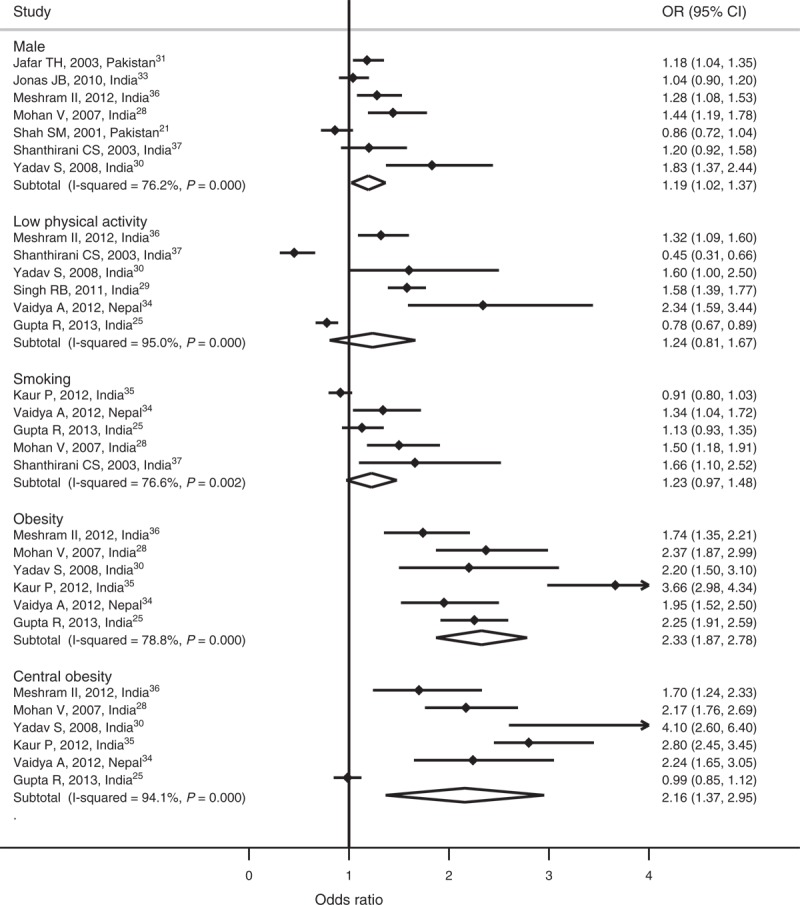
Meta-analysis of risk factors.

Meta-analysis was performed and pooled ORs were calculated for more than 5 studies that reported risk factors. In case of 5 or lesser studies, no meta-analysis was carried out. We observed heterogeneity in all risk factors. The pooled OR from random effect analysis showed that the likelihood of having hypertension was higher among men (OR 1.19; 95% CI: 1.02, 1.37), smokers (OR 1.23; 95% CI: 0.97, 1.48), low physical activity group (OR 1.24; 95% CI: 0.81, 1.67), obese (OR 2.33; 95% CI: 1.87, 2.78), and individuals with central obesity (OR 2.16; 95% CI: 1.37, 2.95). We found pooled OR for men, obesity, and individuals with central obesity statistically significant at 95% CI (Figure 3).

Three studies reported an association between diabetes and hypertension (OR 2.23; 95% CI: 1.52, 3.28^30^; OR 3.42; 95% CI: 2.70, 4.13^25^; OR 1.37; 95% CI: 1.09, 1.72^31^). Two studies showed association between hypertension and family history (OR 2.74; 95% CI: 1.86, 4.03^30^; OR 1.44; 95% CI: 1.19, 1.68^35^), high cholesterol (OR 2.83; 95% CI: 2.32, 3.33^25^; OR 2.08; 95% CI: 1.68, 2.58^28^), and high triglyceride (OR 2.18; 95% CI: 1.75, 2.70^28^; OR 1.93; 95% CI: 1.64, 2.22^25^). Two studies measured the association between alcohol intake and prevalence of hypertension (OR 1.01; 95% CI: 0.74, 1.38^37^; OR 1.42; 95% CI: 1.35, 2.21^25^). Similarly, 2 other studies measured the association between low fruit intake and prevalence of hypertension (OR 1.12; 95% CI: 0.96, 1.30^31^; OR 1.74; 95% CI: 1.44, 2.05^25^). Studies measuring the association of prevalence of hypertension with high fat intake (OR 1.33; 95% CI: 1.11, 1.55)^25^ and high salt intake (OR 1.54; 95% CI: 1, 2.35)^34^ found a statistically significant association.

## DISCUSSION

This is the first comprehensive report to systematically evaluate the scientific literature on the prevalence and risk factors of hypertension in the SAARC region. The prevalence of hypertension based on the most recent publications^16,18,25,31,38–40^ in the countries of the region was: Bangladesh: 17.9%; Bhutan: 23.9%; India: 31.45%; Maldives: 31.5%; Nepal: 33.8%; Pakistan: 25%; and Sri Lanka: 20.9%. The pooled prevalence obtained from our study was 27% ranging from 13% to 48%. A systematic review from the Arab countries found almost similar prevalence of hypertension (29.5%)^46^ and another systematic review conducted for worldwide studies in 2004 reported global prevalence of approximately 30%.^47^

The lowest prevalence was observed in the study^13^ from Bangladesh, which may be explained because of a comparably lower age group of the respondents (mean age = 28.19 years). Our findings showed that the prevalence of hypertension varied between and within the countries. Multiple factors such as age of participants, method of BP measurement, and number of readings of measurements are likely to influence such variation. However, differences in the lifestyle and socioeconomic status of SAARC member states, as well as rural and urban areas within countries also need to be taken into consideration while explaining this variability. Overall, we noticed over 7% difference in aggregated prevalence rates for rural and urban areas. The geographic variations in hypertension prevalence has been well documented.^48^

Majority of studies found the prevalence of hypertension higher among men than among women (combined OR men: 1.19; 95% CI: 1.02, 1.37). Eight reviewed articles showed that the prevalence of hypertension was higher among women than men, which is contrary to the data reported from Sub-Saharan Africa^49^ but compatible with the study from Arab countries.^46^

The reported high prevalence of hypertension in the SAARC region could be because of the epidemiological transition in disease pattern from communicable to noncommunicable diseases.^50^ The increase in the number of people affected by hypertension is attributed to population growth, aging, and the presence of behavioral risk factors such as unhealthy diet, harmful use of alcohol, lack of physical activity, excess weight, and exposure to persistent stress.^51^ For instance, obesity prevalence has reportedly reached epidemic proportions in the SAARC region as shown by a systematic review.^52^ In the past decades, substantial socioeconomic and demographic changes have occurred in the SAARC region resulting in the transition from rural to urban lifestyle. These might be the reasons behind the higher prevalence of hypertension in the urban areas. However, the lack of consistency in these studies allows us only to hypothesize.

Additionally, there was very little information on temporal trends in the prevalence of hypertension. This has restricted our ability to predict whether hypertension prevalence across the SAARC countries is in fact increasing. For example, studies conducted before 2005 also reported high prevalence of hypertension indicating that the recent increase observed might be not only because of actual increase in prevalence of cases but also because of preexisted cases. However, a 3-fold increase in the prevalence of hypertension within 15 years as reported by a Nepalese study indicates that hypertension within this country is rapidly increasing.^34^ Seven countries of this region account for around 15% of the global burden of hypertension (Table 4). The mean estimated prevalence for different regions by WHO lies in between range reported by our study (from 13% to 48%). It is also important to note that SAARC populations are among the youngest populations in the world and life expectancy will definitely increase in coming years.

**TABLE 4:**
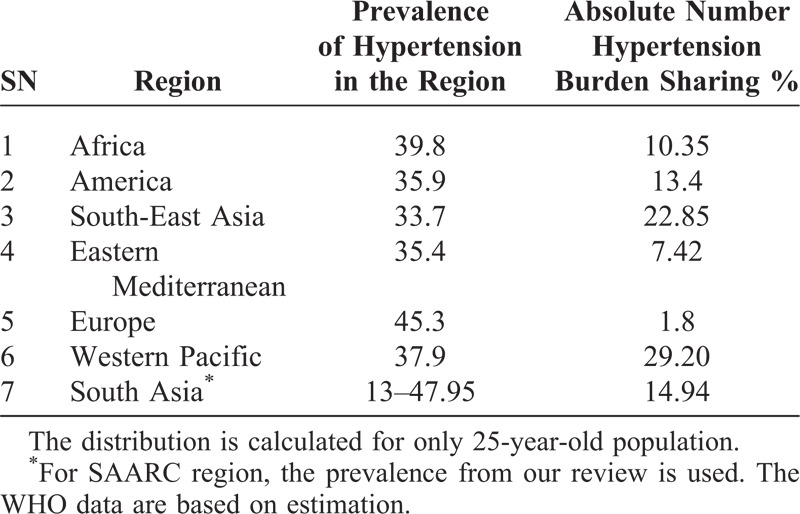
Region-Wise Prevalence and Absolute Number of Hypertension

The study of hypertension is not only important because of its higher prevalence in the SAARC region but also because of the fact that it is one of the most important modifiable risk factors for CVDs. In this meta-analysis, we reported that a number of modifiable risk factors such as obesity, smoking, and physical activity were associated with high prevalence of hypertension in this region. A review on hypertension in developing countries found that several risk factors for hypertension (urbanization, aging of population, change in dietary habits, social stress, high illiteracy rates, poor access to health facilities, bad dietary habits, and poverty) seem to be more common in developing countries than in developed regions.^48^ It should be noted that overall prehypertension (N = 9) was found to be much higher in the urban areas (average of 32.01%, SD = 3.28) as compared to rural areas (average of 23.62%, SD = 5.33). Our observation suggests the need for more systematic reporting of prevalence of hypertension in the future including standardization of measurement and reporting of risk factors.

We observed wide variations in hypertension prevalence across studies and countries of interest. These large differences suggest the importance of examining factors that may contribute to community hypertensive burden, including differences in socioenvironmental status. Studies have previously considered BP variability in the context of social, behavioral, and genetic factors. More specifically factors that have been deemed important include socioeconomic status, dietary intake, race, and epigenetic modifications, which begin early in life and reflect the complex relationship of these genes and environment interactions.^54–57^ A systematic review conducted from India showed that in urban populations exposed to life stress acculturation and modernization, the hypertension prevalence rates have doubled in the last 30 years.^58^ Hypertension variation across country regions suggests that hypertension may be described and defined not as an aggregate of region, but by its diversity within and across a region of interest. Given that the country variability has been observed in our meta-analysis, we suggest that further studies are needed to explore environmental, social, and genetic factors that contribute to within country hypertensive variation.

Despite heterogeneity in different studies, our meta-analysis and systematic review showed that there is a need for prioritizing hypertension for better prevention, diagnosis, and management on the basis of known modifiable risk factors. Primary prevention of hypertension by focusing on the above-mentioned modifiable risk factors is a feasible way to scale up at the population level in this region. A number of interventions such as weight loss programs, dietary sodium reduction, moderation in alcohol consumption, increased physical activity, potassium supplementation, and modification of whole diets have proven effective^59^ against hypertension and could be replicated in the SAARC region as well. Similarly, lessons can be learned from the past experiences of developed countries in Europe, North America, Australia, and New Zealand that showed substantial decrease in age-adjusted cardiovascular mortality after adopting comprehensive approaches over 25 years^60^ in addition to the treatment interventions. Health policies in the region now need to focus on strategies targeting general population as well as high-risk groups such as urban adults.

## LIMITATIONS OF THE STUDY

Age range of study participants made comparisons of the studies difficult. Most of the articles included in our systematic review provided only crude prevalence rates of hypertension and prehypertension. We note, if available, that the age-adjusted prevalence is useful to determine the onset of hypertension across regions. Moreover, when adjustment was made for different variables in the logistic regression, it remained difficult to identify possible confounders. Similarly, the categorization of certain variables such as obesity, smoking, and levels of physical activity had different classifications across reported studies. Our study is limited only to MEDLINE database searches, which may not cover all the studies conducted in this field with the growing expansion of non-indexed local journals, non-English publication, and open access platforms, for example, not captured via MEDLINE. It has also been suggested that including English-only articles in meta-analyses in conventional medicine for chronic diseases such as hypertension does not bias primary findings.^61^ Moreover, we did not perform analyses to identify publication bias of the articles, as it is not relevant in context of prevalence studies.

## CONCLUSION

Our review highlights the high prevalence of hypertension in the SAARC region. There were differences in the prevalence of hypertension and prehypertension in rural and urban areas. The prevalence of hypertension varied from 13% to as high as 48%. Given the lower socioeconomic conditions of the SAARC region, a high burden of underlying hypertension is likely to impact on health systems if overall life expectancy increases.

## ACKNOWLEDGMENTS

The authors would like to thank Aarhus University Library for assisting on article searches. They would also like to thank Ethel Mary Brinda Alexander for providing comments on the draft manuscript.
